# Laryngopharyngeal reflux concept: what is known and what should we focus on?^☆^

**DOI:** 10.1016/j.bjorl.2019.01.001

**Published:** 2019-01-19

**Authors:** Seher Sirin, Ferhan Öz

**Affiliations:** a University of Kocaeli, School of Medicine, Department of Otorhinolaryngology-Head and Neck Surgery, Kocaeli, Turkey; b Acıbadem Bakırköy Hospital, Department of Otorhinolaryngology-Head and Neck Surgery, Istanbul, Turkey

Despite the large number of articles published on laryngopharyngeal reflux (LPR), an overview of the condition reveals many unclear points. Currently there is no “gold standard” for a definitive diagnosis of this very prevalent problem and its consequences. The concept of reflux appears to be present without objective criteria. On the other hand, epidemiological studies on this topic have shown an increase in its incidence.^1^, ^2^ Is it more common in the community and overlooked by patients or physicians? Were patients whom we thought had LPR, in fact, misdiagnosed? Is this one of the reasons, or the main reason, for treatment failure in LPR? While the answers to these questions remain unclear, assessing the relevance of LPR and gastroesophageal reflux disease (GERD) based on what we know will contribute to a better understanding of the LPR concept and its unclear boundaries.

The term gastroesophageal reflux refers to the backflow of gastric contents into the esophagus. Actually, it is a physiological condition. When this condition causes troublesome symptoms and/or signs affecting the quality of life of the patient, we called it pathological reflux. Two main “pathological” conditions are recognized: GERD and LPR.

The term GERD is a clinical directional term that refers to excessive backflow rising (from the stomach to the esophagus) that causes esophageal tissue damage (esophagitis) and/or clinical symptoms (heartburn, regurgitation), also called typical reflux disease or heartburn reflux. The other main pathological condition, LPR, is a locational term meaning that refluxed material causes symptoms and/or signs into the laryngopharynx by different mechanisms.

Numerous synonyms for LPR have been defined. The first known term was reflux-related laryngeal disease (reflux laryngitis), reported by Cherry in 1968.^3^ Currently, the most widely used term is LPR, adopted by the American Academy in its 2002 Position Statement.^4^ The other common term, which is better understood by patients, is heartburn reflux.

These two conditions are different from each other, with different symptoms and different mechanisms, which are as follows: GERD is more related to LES and esophageal defense mechanism incompetency, and LPR is more related to UOS and, according to new data, reactivated pepsin.^5^ Even more importantly, patients with severe esophagitis may not have accompanying LPR diseases or vice versa: patients with severe LPR may not have GERD. Scientific evidence shows that LPR is not an advanced stage of GERD. However, one third of LPR patients have GERD.^6^ This made us think that there might be a causal link between them. In those patients, does GERD cause LPR? Alternatively, are they concomitant diseases with similar underlying mechanisms, and is GERD a co-factor? Is another mechanism responsible for LPR in patients who already have GERD, and their co-occurrence is just coincidence? These questions remain unclear. However, one thing of which we are sure is that the laryngeal mucosa is more acid-sensitive than the esophagus. While 50 acid exposures per day is the upper limit for esophageal injury, experimental studies have shown that 3 acid exposures per week can lead to damage to the larynx! Cell damage occurs in the epithelium of the esophagus when the pH is below 4 but occurs in the laryngeal epithelium at a higher pH and with short-term exposure.^7^ For this reason, LPR should be treated more aggressively and for longer than GERD. The accepted view today is that, although the relevance between them is not fully understood, it is necessary to consider them as different entities and treat them differently; therefore, LPR and GERD are different concepts under the main heading reflux disease.

For proper treatment, it is necessary to have an accurate diagnostic algorithm. What should be our next step for a patient who has typical LPR symptoms with a supportive laryngeal examination? Should we refer for further evaluation? If any alarming symptom is present, absolutely yes; if there is unresponsiveness to treatment, yes. But for a definitive diagnosis, should we refer?

Double probe ambulatory pH monitoring was accepted as the gold standard technique for LPR diagnosis for many years. However, a negative result on pH monitoring cannot be interpreted as the absence of LPR: 3 reflux attacks per week can damage the laryngeal epithelium, but standard pH monitoring lasts for 24 h so the evaluation period is inadequate for LPR diagnosis as the patient may not have been symptomatic on the examination day. The degree of symptoms and signs are not correlated with test results, even in patients with a positive result on pH monitoring, and is insufficient for evaluating the response to treatment. On the other hand, the pH monitoring test shows only acid reflux.

Multichannel Intraluminal Impedancemetry is superior in this respect: it reveals acid, weak acid, and nonacid reflux and helps us determine our treatment strategy. But, we know that retrograde movement is not the only cause; esophageal hypersensitivity, hypervigilance, psychosocial factors, or just a lifestyle change and diet taken (i.e., acid juices) that pass through the laryngopharynx can result in LPR. Regarding all these costly diagnostic difficulties, the most reliable diagnostic method for LPR is still remission of symptoms and signs by lifestyle changes as well as anti-reflux treatment.

Recent studies have focused on overdiagnosis of LPR, especially in dysphonic patients. Thomas et al. reported muscle tension dysphonia, bowing, nodules, and neurogenic diseases as the most common definitive diagnoses following further evaluation for unresolved hoarseness in previously diagnosed LPR patients.^8^ Sulica et al. reported phonotraumatic lesions, neurologic disorders, and age-related changes for patients presenting for a second opinion evaluation previously diagnosed LPR alone.^9^ When LPR is suspected in a dysphonic patient, flexible laryngoscopy and laryngovideostroboscopy should be performed prior to any treatment, as a differential diagnostic tool to prevent and avoid misdiagnosis as well as unnecessary treatments. Thus, LPR should be considered a diagnosis of exclusion in the presence of dysphonia.

Proton pump inhibitors (PPIs) are the main therapeutic agents for reflux disease. PPIs are fast, strong, and long-acting drugs. The most important factor affecting their treatment success is their improper use. The patient should be questioned about drug use to reveal possible inappropriate use. More than half of patients do not take their PPIs within 1 h of breakfast.^10^ PPIs require expression of the proton pump along the parietal cell canaliculi membrane, which means that they should be taken 30–60 min before the meal. Again, LPR therapy should be more aggressive and longer. In order to avoid the rebound effect, it is important to remember to reduce the PPI dose gradually before ceasing treatment. Additional treatment with histamine-2-receptor antagonists as a night dose might be beneficial for nocturnal symptoms and should be added intermittently, considering the development of tolerance.

The most common symptom of LPR is dysphonia. Ironically, dysphonia is also the most common persistent symptom even after treatment. LPR affects laryngeal behavior that leads to vocal hyperfunction with either direct or indirect effects. A mucosal edema and hyperemia on the vocal folds occurs as a result of chemical irritation from refluxed material. The load of the vibrating vocal fold increases. It does not change the vibration of the vocal folds but leads to laryngeal behavioral change with time and muscle behavior change. Reflux disease and upper respiratory infection are well known as the most common triggers of functional voice disorders. Even if the symptoms from an underlying cause improve with medical treatment and lifestyle change, this altered laryngeal behavior might be causative of persisting symptoms (dysphonia, throat clearing, and chronic cough). Therefore, for refractory LPR patients’ symptoms, management should be considered multidisciplinary and include voice therapy.

Reflux is a chronic and intermittent disease. Therefore, whether the patient is asymptomatic or symptomatic, whether medical treatment is initiated, or the surgical treatment is applied, lifestyle and dietary change in the reflux patient take place in the first step and persist until the last step.

For refractory cases with persistent signs of reflux, a higher dose of PPI therapy typically provides better outcomes. In some selected patients with severe complications or those on continuous PPI, a laparoscopic anti-reflux surgery is a promising method.

Last of all, the entity of the LPR concept is undeniable. But we are still far from the point of having objective criteria. Apparently, some conditions with similar symptomatology occurring in or affecting the laryngopharynx may overlap with LPR as a cause, consequence, or associated factor. This fact leads to diagnostic and therapeutic failures. Therefore, establishing a multidisciplinary collaboration between an otolaryngologist, gastroenterologist, speech/language therapist, and gastroenterological surgeon will provide a comprehensive approach with a view to developing reliable and acceptable diagnostic as well as treatment modalities for the LPR concept.

## Conflicts of interest

The authors declare no conflicts of interest.
